# Iatrogenesis in the Context of Residential Dementia Care: A Concept Analysis

**DOI:** 10.1093/geroni/igac028

**Published:** 2022-04-21

**Authors:** Patricia Morris, Rose McCloskey, Donna Bulman

**Affiliations:** School of Graduate Studies, University of New Brunswick, Saint John, New Brunswick, Canada; Geriatric Medicine Clinic, Horizon Health Network, Saint John, New Brunswick, Canada; Department of Nursing and Health Sciences, University of New Brunswick, Saint John, New Brunswick, Canada; Faculty of Nursing, University of New Brunswick, Fredericton, New Brunswick, Canada

**Keywords:** Cognitive impairment, Nursing homes, Patient harm, Patient safety

## Abstract

**Background and Objectives:**

This concept analysis aims to explore iatrogenesis within the context of residential dementia care and to distinguish this phenomenon from similar phenomena, such as abuse and inadvertent harm.

**Research Design and Methods:**

Walker and Avant’s method for concept analysis was used to define critical attributes of iatrogenesis within residential dementia care, and to explore antecedents and consequences of its occurrence. A review of the literature about iatrogenesis in the context of residential dementia was conducted across 4 electronic databases. Texts about iatrogenesis in surgery, medicine, social work, psychology, and other relevant disciplines were also reviewed to provide additional context for the concept.

**Results:**

Iatrogenesis takes a unique form in residential dementia care. The final definition of the concept proposed in this article is habituated, forceful, hands-on care provided to residents who exhibit responsive behaviors that result in emotional, physical, spiritual, social harm, and/or gradual functional decline, that is provided with the intention of supporting the resident’s safety and dignity.

**Discussion and Implications:**

The definition of iatrogenesis proposed in this article is an initial step toward developing evidence-based practice for the provision of nonconsensual assistance in residential dementia care. A theoretical definition like the one proposed in this article may serve as a starting point for the operationalization of the concept, which would promote future empirical research into staff and residents’ experiences of health care-inflicted harms in this context. Theoretically, it contributes to critical conversations about the narratives, myths, and misperceptions that facilitate the provision of nonconsensual care.

**Translational Significance:** Reconceptualizing iatrogenesis as a relevant concept in nursing homes, and as a negative outcome of unwanted care, offers the potential to help disrupt current notions of how best to care for residents and to protect them from harm. Considering the possibility of iatrogenesis within the context of nursing homes may motivate staff to embrace new person-centered care programs.

Adults with a dementia diagnosis are often admitted to residential aged care facilities (RACFs) such as nursing homes and psychogeriatric institutions due to health and safety concerns in the community (Campbell-Enns et al., 2020). They may be admitted to prevent or reduce harms that result from forgetfulness, wandering, falls, medication self-administration errors, and general functional decline (Douglas et al., 2011). They may also be admitted due to concerns about self-neglect. Self-neglect is common among older adults with dementia, although the exact prevalence is unknown (Qian et al., 2021). Unintentional self-neglect in the context of dementia is characterized by a new or worsening inability to perform essential activities of daily living related to food, hygiene, living environment, and personal safety that were performed before the onset of dementia. It might include, among other things, improper toileting, reduced or ineffective bathing, hoarding or improper home maintenance, or an inability to prepare adequate meals. Self-neglect (also known as Diogenes syndrome and by the pejorative term “senile squalor syndrome”; Papaioannou et al., 2012, p. 187), poses a serious threat to older adults’ health and psychosocial well-being (Dong, 2017). Self-neglect places older adults with dementia at risk for early mortality, and it can have deleterious effects on overall health and well-being if left unaddressed (Qian et al., 2021). It often necessitates admission to a skilled nursing facility where essential care can be provided, but the provision of this care is often rife with challenges (Dong, 2017).

Primary goals of residential dementia care include promotion of functional ability and, where possible, maximal independence (World Health Organization [WHO], 2015). There are, however, times when residents resist or refuse care that is essential to their overall well-being. In cases where a resident’s cognitive impairment predisposes them to self-neglect, for example, staff cannot simply stand by and watch a person deteriorate. A wealth of literature has explored the tensions staff experience as they attempt to balance respect for autonomy (the resident’s right to self-rule), with ethical commitments to nonmaleficence and beneficence (the professional responsibility to prevent harm and promote the resident’s greatest good; Lejman et al., 2013; Morris et al., 2021; Nilsson et al., 2016; Smebye et al., 2015; Svendsen et al., 2017; Tranvåg et al., 2013; van den Hooff & Goossensen, 2015; Vandrevala et al., 2017). Many training programs, such as the popular *Gentle Persuasive Approaches* (Advanced Gerontological Education Inc., 2021) and *Bathing without a Battle* (Barrick et al., 2003), teach care providers approaches that lessen resistance to care and help decrease distress in situations where care must be provided against the resident’s expressed will. These programs are rooted in the person-centered care approach, which emphasizes the continued humanity of people with dementia and argues that all behavior (no matter how challenging) has meaning (Cohen-Mansfield et al., 2015). Through the lens of person-centered care, resistive behaviors such as striking out, swearing, biting, and other common responses to unwanted care become intelligible as responsive behaviors that express an unmet need (Cohen-Mansfield et al., 2015). At times, it is possible to uncover the unmet need and tailor the approach or plan of care to meet that need. As past and present nurses in RACFs, though, the authors are all too aware that there are times when necessary care is rendered through the use of “loving force” (Olofsson, 1995, p. 326) by care providers who must take responsibility for the well-being of residents who are incapable of caring for themselves.

Even when the care provided is necessary and the effort used to provide it is loving, residents may be at increased risk of emotional, physical, and social damages. Research about unwanted care in acute psychiatric wards reveals that receiving unwanted care can have serious and long-lasting impacts on well-being (McGuinness et al., 2018; Vuckovich & Artinian, 2005; Wormdahl et al., 2021). Similar research, which explores residents’ experiences of unwanted dementia care, is needed to understand the unique impacts of unwanted care in this context. In psychiatry, unwanted care is often rendered short-term, in hopes of facilitating a person’s recovery and return to independence (McGuinness et al., 2018). In the context of (currently irreversible) neurodegenerative changes, unwanted care often occurs long-term and without hope of curing underlying dysfunction. While much has been written about strategies to reduce or manage refusals of care in dementia, research is still in its infancy about the subjective experiences and objective impacts of repeated, long-term provision of unwanted care to people living with dementia (Backhouse et al., 2020). To encourage future research about the impacts of unwanted dementia care, this article proposes a theoretical starting point for the conversation by offering a provisional definition of iatrogenesis in the context of residential dementia care. We propose that, despite loving intentions, the provision of unwanted care may well lead to iatrogenic emotional, physical, and psychosocial damages that warrant further research and practice reform. This article proceeds using Walker and Avant’s (2019) method for concept analysis to define the critical attributes of iatrogenesis in this context, along with the antecedents and consequences of its occurrence. We begin with a discussion of the background literature on patient safety and adverse health care events to situate our analysis, and then we move into a description of methodology and the results of our analysis. We conclude with a discussion of future directions in research that might be opened up by the theoretical definition of iatrogenesis we offer in this article.

## Background

Literature on patient safety and health care-linked harms gained traction in the late 1990s and early 2000s, with the publication of several alarming reports that detailed a significant difference between what is done and what is theoretically obtainable in health systems around the world. The Institute of Medicine’s (IOM) “To Err is Human” report (2000), for example, estimated that 44,000–98,000 patients die each year from preventable errors in hospitals across the United States. Twenty years after the publication of the IOM’s report, the WHO (2019) estimated that as many as four in 10 patients continue to be harmed through interaction with global health care systems and that 80% of this harm is preventable. As the complexities of health care systems and technologies increase, the “burden of harm due to unsafe care” shifts increasingly onto the patient (WHO, 2019, para. 6). Globally, the most common safety situations causing concern are medication errors, health care-associated infections, unsafe surgical care procedures, unsafe injection practices, diagnostic errors, unsafe transfusion practices, radiation errors, undiagnosed sepsis, and venous thromboembolism (WHO, 2019). Some harms result from procedural issues, such as a provider’s failure to perform to the necessary standard of care, while others result from providers’ failures to apply the best available evidence (Higham & Vincent, 2021). Still others result from structural issues, such as understaffing and inadequate skill mix, which result in preventable delays and errors (Higham & Vincent, 2021). The dichotomy between individual and structural perpetrators of harm is, however, increasingly recognized as false in the patient safety literature (Higham & Vincent, 2021; IOM, 2001; Lark et al., 2018; WHO, 2019). In nearly all situations where an error can be linked to an individual provider or team, there is a health care system that allowed that harm to occur because of “fundamental shortcomings in the ways care is organized” (IOM, 2001).

A variety of safeguards have been put in place at global, national, and institutional levels to address these shortcomings in the ways care is organized. For example, in 2008 the WHO released a “Safe Surgery Saves Lives” checklist of safety verifications that guard against procedural and individual errors throughout the operative process. This involves, among other safeguards, marking the surgical site while the patient is still awake to minimize sentinel events such as the removal of the wrong limb or organ. On an institutional level, health care facilities have also trialed targeted quality improvement projects to address the systemic causes of health care-related errors. For example, many institutions have opted to document near-miss events in hopes of learning from mistakes and preventing future errors through systems-level changes (Crane et al., 2015). Targeted initiatives that address specific and well-known patient safety concerns have also largely been quite successful (e.g., Kelley et al., 2018), while broader initiatives to improve patient safety across the care continuum have seen significantly less success (Lark et al., 2018). In this article, we shift our focus away from specific time-limited errors that result in measurable physical harms (e.g., nosocomial infections, adverse drug reactions, and medication errors). Instead, we turn our attention to a kind of harm that befalls individuals with dementia who repeatedly require seemingly “routine” care that they do not wish to receive. We argue that this harm—which we will define as iatrogenesis in this context—results in less tangible (but no less frightening) damages.

## Method

Concept analysis aims to clarify the definition of an abstract concept so that it might be used to guide future theorizing and empirical research (Walker & Avant, 2019). While there are many approaches to concept analysis, the authors rely on Walker and Avant’s (2019) approach for this work. Walker and Avant’s approach draws on the work of philosopher John Wilson (1963), who was among the first scholars to argue that ambiguous concepts require denotational analysis to be useful for critical thought. Walker and Avant have been criticized for borrowing Wilson’s approach without paying adequate attention to the philosophical underpinnings of his work (Risjord, 2009). Their method has also been criticized for its unsystematic approach to literature searches, and for its lack of attention to contexts in which a concept circulates (Weaver & Mitcham, 2008). Walker and Avant have noted these critiques (2019, p. 185) but have offered little in the way of rebuttal to them. To support our use of the method, we draw attention to the numerous studies that have advanced nursing knowledge through its application (e.g., Abdolrahimi et al., 2017; Altmann, 2008; Johnston et al., 2014; Sierra & Cianelli, 2019; Taylor et al., 2017). These analyses are indications that, while imperfect, the method continues to be useful for the advancement of nursing knowledge.

Walker and Avant (2019) propose proceeding through six steps: (a) identify the concept and its uses; (b) determine defining or critical attributes; (c) identify a model case; (d) identify borderline, related, contrary, invented, and/or illegitimate cases; (e) identify antecedents and consequences; and (f) define empirical referents. These steps are intended to be iterative, meaning that at each stage of analysis the previous stages may require revision because of ideas that arise from a later stage. While they are described in sequence here, the actual analysis took a much more winding route.

**Figure 1.**
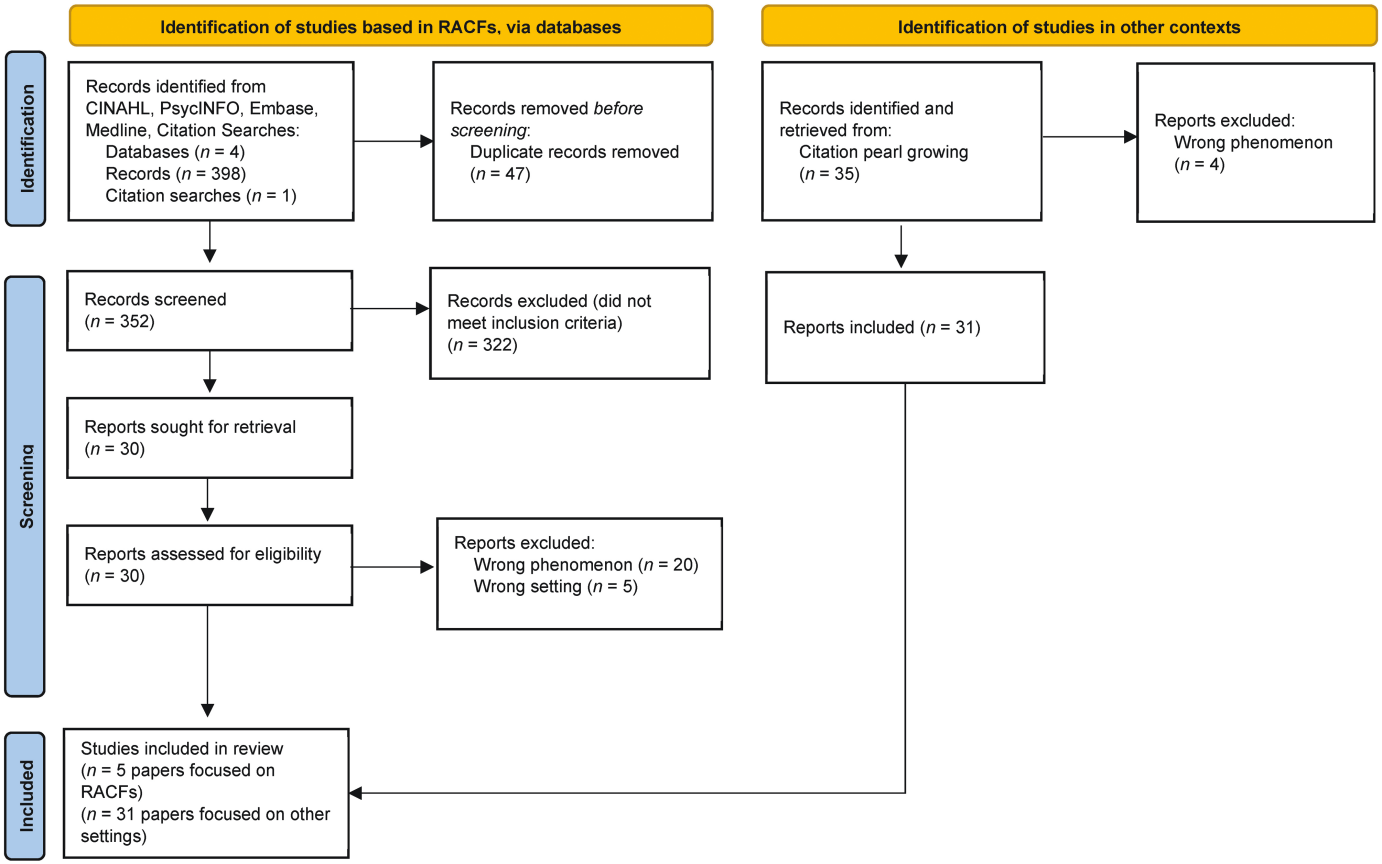
PRISMA flow diagram. RACF = residential aged care facility.

To begin our analysis, we conducted a systematic review of the literature about iatrogenesis in the context of residential dementia care in the following databases: CINAHL (EBSCO), PsycINFO (EBSCO), Embase (Ovid), and Medline (Ovid). The search strategy for this search is included in Appendix 1. The full results of the search are detailed in the PRISMA flow diagram (Figure 1). The search returned 351 articles after duplicates (*n* = 47) were removed. The citations were uploaded into Covidence (Veritas Health Innovation), and titles and abstracts were reviewed independently by the first and second authors. Disagreements were resolved through conversation. Studies were considered for inclusion if they were written in English and focused on harms that result from personal care, including hygiene care, assistance with nutrition, and assistance with other activities of daily living. Only studies set in RACFs were considered for inclusion at this stage. In cases where the setting or focus of care was unclear, the article was moved forward to the full-text review. Thirty full-text articles were retrieved and assessed for inclusion by two independent reviewers. Articles were excluded if they took place in the wrong setting (*n* = 5), or if they did not focus on harms from personal care (wrong phenomenon *n* = 20).

These included articles about adverse drug reactions, adverse effects from participation in research, and falls. To complete the section on critical attributes of the concept as it is used in other contexts, we reviewed articles on iatrogenesis in medicine and social services. We retrieved these texts through citation pearl growing, using articles that described the concept’s evolution in medicine (Sharpe & Faden, 1998) and the social sciences (Loeb & Slosar, 1974) as pearls. We retrieved 35 full-text articles in total, and we excluded four texts along the way as they explored the wrong phenomenon. We continued our search until the authors agreed that saturation had occurred and there were no new insights gleaned from continued review of the literature (*n* = 31).

## Results

### Step 1: Identify the Concept and Its Uses

The first step in Walker and Avant’s (2019) approach is to identify varied uses of the concept under scrutiny. Following from the method’s origins in linguistics, this step helps authors identify how the term is used and its connotations across contexts. This process typically begins with identifying dictionary definitions and etymology and then exploring varied uses of the concept.

In the Merriam-Webster online dictionary, “iatrogenesis” is broadly defined as the process by which an injury or illness is “induced inadvertently by a physician or surgeon or by medical treatment or diagnostic procedures” (para. 1). Taber’s Medical Dictionary (2021) defines the term “iatrogenic” as an adjective describing “any injury or illness that occurs because of medical care” (para. 1). Cambridge English Dictionary (2021) similarly defines “iatrogenic” as an adjective describing diseases or health problems “caused by medical treatment or a doctor” (para. 1). “Iatrogenesis” comes from the Greek words “iatros,” meaning physician and “genesis,” meaning origin or source (Merriam-Webster, 2022).

The concept is used both implicitly and explicitly. Implicit uses are those that seem to reference the concept but are not expressed plainly, while explicit uses refer to the concept directly. In our hunt for implicit uses of the concept, we found several sources that argued that iatrogenesis existed before it was explicitly named as such. Several scholars trace the roots of iatrogenesis as far back as Florence Nightingale (Larsen, 1989; Sampson et al., 2005), arguing that Nightingale’s focus on handwashing, sanitation, and environmental conditions might be considered an early recognition of health care providers’ capacities to harm through intervention. In their historical look at iatrogenesis, Sharpe and Faden (1998) highlight the American Medical Association’s 1847 Code as a potential early implicit usage due to its focus on physicians’ capacities to “discourage the patient and to depress his spirits” (as cited on p. 61). While these early implicit usages may have set the stage for modern recognitions of iatrogenesis, this liberal reworking of history to give name to a concept not yet coined gives significantly more credit to medical professionals for championing the struggle against iatrogenic damages than perhaps they are due. The historical evolution of the explicit term and its’ definitions provide more insight into the concept’s contentious journey since its introduction.

The term “iatrogenesis” was first used explicitly in medical literature in 1924, in a textbook on psychiatry penned by Professor Eugene Bleuler (1924/2019). According to Sharpe & Faden (1998), iatrogenesis for Bleuler was primarily related to the “suggestibility of the patient” (p. 61). At the height of Bleuler’s influence, the focus was on the power of the physician’s word and the vulnerability of the patient whose cognition or coping was impaired (Sharpe & Faden, 1998; Andrews, 2004). Iatrogenesis occurred when patients experienced negative psychological responses to physicians’ diagnoses (Sharpe & Faden, 1998). In 1960, Chapman coined the term “psychiatrogenic” to describe the exacerbation of clients underlying “personality problems” due to psychiatric intervention (p. 873). After the term’s introduction in psychiatry in the 1920s, the phenomenon was largely ignored in medical literature until the late 1950s and early 1960s (Andrews, 2004).

The conversation on medical iatrogenesis was taken up outside of psychiatry after World War II, with the advent of penicillin and other advanced pharmacotherapies (Sharpe & Faden, 1998). Penicillin was prescribed incorrectly and at alarming rates. As many patients experienced adverse reactions to treatment, health care professionals began to take a renewed interest in the potential harms of medical intervention (Sharpe & Faden, 1998). The term was reintroduced by physicians Barr (1955) and Moser (1956) and was described as a regrettable but unavoidable consequence of medical progress. In particular, Barr (1955) noted that iatrogenic diseases “would not have occurred if sound therapeutic procedure had not been employed” (as cited in Steel, 2004, p. 76). In other words, iatrogenic illnesses were understood as unfortunate risks of vital advancements in drugs, therapies, and techniques that improve well-being and community health.

The definitions of iatrogenesis offered outside of medicine have largely followed a different pattern. Iatrogenesis first became a subject of conversation in the social sciences and humanities when it was taken up by philosopher Ivan Illich. Illich believed that mass education and modern medicine were the root causes of poor quality of life and ill health in the modern world (Wright, 2003). For Illich, the medical establishment’s insistence on pathologization and orientation toward “cures” for nearly all conditions led to a collective inability to cope with hardships and an overreliance on individualized medical services to address structural concerns (Illich, 1976). He viewed modern medical treatment as the root cause of many illnesses, stating that “an expanding proportion of the *new* burden of disease of the last fifteen years is itself the result of medical intervention in favor of people who are or might become sick. It is doctor-made, or *iatrogenic*” (p. 14). For Illich, iatrogenesis was an example of the supreme risks that medicine posed to health and wellness.

This sentiment was later incorporated into definitions of iatrogenesis offered by service-oriented disciplines like Social Work. The term “sociatrogenic” (Loeb & Slosar, 1974) evolved to describe the negative consequences of social service interventions, as researchers began questioning the adage that “doing something is better than doing nothing at all” (p. 52). Challenging this adage required service providers to consider that their care may not always benefit the recipient.

### Step 2: Identify Critical Attributes

The next step in Walker and Avant’s (2019) approach is to identify critical attributes of the concept. Critical attributes are those defining characteristics that “help you and others name the occurrence of a specific phenomenon as differentiated from another similar or related one” (Walker & Avant, 2019, p. 169). Critical attributes are those characteristics that are essential to the concept, without which the presence of the concept is unrecognizable. Walker and Avant (2019) do not, however, offer step-by-step guidance on how critical attributes ought to be intuited. To identify critical attributes of iatrogenesis in the specific context of residential dementia care, we followed the example set by Johnston et al. (2014) and compared and contrasted literature on iatrogenesis in modern interdisciplinary contexts with literature on this topic in the specific context of interest. We compiled a list of all attributes identified in the saturated sample of interdisciplinary literature and compared them with attributes identified in the residential dementia care literature. This helped us arrive at a set of critical attributes that retained the sense of the broader literature but were specific to the context of residential dementia care.

#### Attributes of iatrogenesis in the multidisciplinary literature

The multidisciplinary literature defines iatrogenesis using varying combinations of the following attributes: (a) an adverse consequence of necessary treatment, (b) a consequence of client behaviors or conditions, (c) rare, (d) facilitated by systemic issues, and (e) perpetrated by a health care provider whose practice is deviant or whose assessment skills are inadequate (Table 1).

**Table 1.** Attributes in Multidisciplinary Papers (*N* = 31)

Study	Adverse consequence of necessary treatment	Rare	Related to client’s condition or behaviors	Deviant practice or improper interventions	Systemic issue
Barr and Kauffman (2014)			X	X	X
Cameron and Sheppard (2006) ^a^				X	X
Caplan and Caplan (2001)	X			X	X
Cummings (1979)				X	X
Day et al. (2014) ^a^					X
De Jongh et al. (2008)			X	X	
Decou and Schumann (2018)		X			
Engle et al. (2010) ^a^			X	X	
Fairley et al. (2020)	X	X			
Gatti et al. (2009) ^a^				X	
Gomes et al. (2017)	X				
Hatton et al. (2006) ^a^				X	X
Kimber and Sandell (2009)	X	X	X		
Lim et al. (2020)	X		X		
Lui et al. (2020)	X				
Lyons (2006)	X			X	
Meessen et al. (2003) ^a^					X
Micheletti et al. (2020)	X				
Moynihan (1993) ^a^					X
Nariai et al. (2020)	X				
Peer and Shabir (2018)	X			X	X
Reynolds and Crea (2015) ^a^				X	
Rothstein et al. (2000) ^a^					X
Ruffino et al. (2020)	X				
Schultz and Preston (1983) ^a^				X	
Shapiro et al. (2019)	X			X	X
Sheridan (1989)		X	X	X	
Suzuki et al. (2020)	X				
Sweetwood and Sweetwood (2001) ^a^				X	X
Wallace et al. (2007)				X	
Ziegler (2010)			X	X	X
Total	*n* = 13	*n* = 4	*n* = 6	*n* = 17	*n* = 12

^a^Indicates article has a nonmedical or primarily psychosocial focus.

The notion that iatrogenic damages were unfortunate but largely unavoidable adverse consequences of necessary treatment was present in 13 of the 31 articles (41.9%). All of these articles described medical care and they presented iatrogenic damages as “potentially life-threatening complications” (Ruffino et al., 2020, p. 1) of necessary surgeries, a “vessel injury” sustained during tumor resection (Nariai et al., 2020), an “adverse clinical condition” (Gomes et al., 2017, p. 511) that results from the provision of many types of care, an “unsolved drawback” of revolutionary new investigations (Micheletti et al., 2020, p. 1) and as “another pattern of vascular trauma” similar to blunt or penetrating traumas that might occur in the community (Lui et al., 2020, p. 5). Sharpe and Faden (1998) identify this tendency to describe iatrogenesis as an unavoidable consequence of progress as a “utilitarian understanding” (p. 64). They note that, historically, “investigations of iatrogenic illness reflected a firm confidence in medicine and its practitioners despite the documented extent of complications” (p. 64). This same confidence is apparent in many of the studies included in this analysis.

In a small number of cases (*n* = 4; 12.9%), iatrogenic damages were described as rare complications. For example, Kimber et al. (2009) describe complications occurring in only a small subset of the treated population, while Fairley et al. describe the incidence of nosocomial colonic gas gangrene after colonoscopy as a “rare but documented complication” (Fairley et al., 2020, p. 3). Lim et al. (2020) even describe an iatrogenic cervical spine break from gradual cervical traction as a “fortuitous complication” (p. 3) that meant the client did not require further surgeries. One study by Decou and Schumann (2018) explored potential iatrogenic effects of performing suicide assessments and concluded that iatrogenic suicidal ideation is extremely unlikely to arise from these assessments. They argue that fears of iatrogenesis in suicide assessment are overinflated.

Six articles (19.4%), which included both medical and social studies, identified specific client characteristics or behaviors as (one of) the primary reasons for iatrogenesis. For example, Lim et al. (2020) describe advanced age and preexisting bone disease and infection as risk factors leading up to an iatrogenic cervical spine break in their patient. Ziegler (2010) notes that common activities that attract lesbians (e.g., clubs, music festivals, and sporting events) predispose this population to drug use. They argue that this can impact their susceptibility to iatrogenic addiction. Barr and Kauffman (2014) note that clients older than 65 are twice as likely to experience iatrogenic harms during routine medical treatments.

Care provider mistakes and deviant practice are noted in 17 of the included articles (56.7%). While some authors noted that mistakes can be made by any care provider regardless of competency level (Barr & Kauffman, 2014; Caplan & Caplan, 2001), others pointed to providers’ inattentiveness to patients’ cues as precursors to iatrogenesis (De Jongh et al., 2008; Sheridan, 1989; Ziegler, 2010) and their use of the wrong intervention as the root cause of damages (Cameron & Sheppard, 2006; Caplan & Caplan, 2001; Day et al., 2014; Engle et al., 2010; Lyons, 2006; Schultz & Preston, 1983; Wallace et al., 2007). In one case, authors pointed to a physician’s altered level of consciousness so severe that a patient was stripped of legal rights to make her own decisions (Sweetwood & Sweetwood, 2001). Iatrogenic effects were also identified as the direct result of clinicians’ mislabeling of clients. For example, Schultz and Preston (1983) completed a survey in West Virginia that explored children’s experiences of sexual abuse. They argue that social service providers and mental health professionals need to consider the iatrogenic trauma that might follow from labeling an experience as a trauma for the client who did not experience it as such.

In many cases, in addition to individual actors, systems were implicated as causes of iatrogenic harms in the broader literature. Systemic causes were noted in 12 articles (38.7%) and included overarching school disciplinary practices (Cameron & Sheppard, 2006), juvenile legal practices (Gatti et al., 2009), prison conditions (Hatton et al., 2006), federal illicit drug policies (Moynihan, 1993), sex offender registries for offenders who perpetrated minor crimes (Day et al., 2014), and funding structures for mental health care (Rothstein et al., 2000). In a particularly scathing critique of federal drug policies in the United States, Moynihan (1993) states: “clearly federal drug policy is responsible for a degree of social regression for which there does not appear to be any equivalent in our history” (p. 362). Relatedly, and more than 10 years later, Hatton et al. (2006) argued that the living conditions of women in U.S. prisons are a frequent source of poor health. Caplan and Caplan (2001) identify iatrogenesis as a concept “that originated in medical practice to denote the damage induced in a patient as a by-product of a therapeutic intervention” (p. 1) and seek to extend this usage of the concept to apply to any intervention (individual or systemic) which has the “declared intention of curing or preventing psychosocial disorders” (p. 1). The focus on *system-as-perpetrator* is a common theme that runs through much of the multidisciplinary literature. This is consistent with the focus on systemic causes in the patient safety literature.

#### Critical attributes of iatrogenesis in residential dementia care

Residential dementia care straddles the line between health and social care, making it an interesting site for an exploration of iatrogenesis. We examined the five articles about iatrogenesis in residential dementia care for the presence of the five attributes identified in the wider literature previously. We then modified and extracted additional attributes as needed to reflect the character of this body of literature. This process revealed three critical attributes of iatrogenesis in residential dementia care: (a) it is related to a client’s overt behaviors, (b) it is an adverse outcome of necessary care, and (c) it is habituated.

The literature about iatrogenesis in dementia care focuses on clients’ cognitive capacities and responsive behaviors that challenge health care providers in significant ways. For example, Chandler et al. (2018) note that staff, institutions, and policies often prioritize a resident’s rationality in determining their freedom to choose in a range of situations. On noting a lack of rationality in residents’ decision-making, staff may shift from acknowledging residents as “holders of rights” and instead view them as “subjects of welfare or protectionist approach(es)” (p. 477). Travis et al. (2003) note that high rates of pain among older adults with dementia, alongside their decreased or nonpreferred expressive abilities (e.g., screaming vs. calm explanation), set the stage for iatrogenic pain caused by hands-on care. Cotter (2005) emphasizes that residents’ impaired memories, “cognitive declines in language, judgment, and visual perception” (p. 81), and “behavioral symptoms such as agitation, anxiety, psychosis, or pacing” (p. 81) act as catalysts for health care decisions enacted by force. Iatrogenic damages in residential dementia care, then, are not experienced equally by all residents. Instead, those residents who exhibit “challenging behaviors” (Chandler et al., 2018, p. 465) are at greater risk for receiving care by force.

Much like the broader literature, this subset of articles describes iatrogenesis as an adverse outcome of necessary care. The reasons for this care provision are, however, significantly different than those offered in the broader literature. All five articles note that best practice evidence for prevention of iatrogenesis already exists for residential dementia care, but that it is not applied consistently (Chandler et al., 2018; Cotter, 2005; Dudevich et al., 2018; Hofmann & Hahn, 2014; Travis et al., 2003). This may be because the resident does not respond to other best-evidenced approaches (Cotter, 2005), or because staff members are unaware of alternative approaches (Travis et al., 2003). Three articles argue that staff justify their actions as attempts to promote dignity and safety which require them to work outside of established best practices (Cotter, 2005; Dudevich et al., 2018; Hofmann & Hahn, 2014). Travis et al. (2003) note that iatrogenesis is an adverse outcome of care that is necessary but performed in ways that do not adhere to the best available evidence because staff have not been trained in other approaches. Chandler et al. (2018) note more broadly that a “welfare approach” (p. 472) is typically undertaken in work with people with cognitive impairments, which enables care providers to justify treatment in the name of charity or care of the cognitively impaired person. In this case, the necessity of forceful care performed against the will of the client is justified by prioritizing the resident’s dignity and safety. Iatrogenic damages result from care that staff believe must be provided, despite the harm it does, to support safety and dignity.

The habituated nature of iatrogenesis in residential dementia care is clear from the ways that all authors described forceful care provision as nearly ubiquitous and routine. While all authors argued against the provision of unwanted care, each highlighted the ways that this care has become habitual for staff. That staff is habituated to unwanted care provision is clear, for example, in Travis et al.’s (2003) description of staff members who have not been told to avoid inflicting pain, Cotter’s (2005) long list of potential harms that befall residents at the hands of staff, Chandler et al.’s (2018) description of widespread and unregulated provision of forceful care, and Hofmann and Hahn’s (2014) description of widespread restraint usage. All five articles identified iatrogenic damages as so habitual that they create an environment of risk for injury for residents. For example, Dudevich et al. (2018) describe iatrogenic harms as the “unintended occurrence of harm” (p. 81) that simultaneously increases older adults’ risk for future harm. Cotter (2005) describes iatrogenic outcomes as “increased *risk for* falling, pressure ulcers, incontinence, muscle deconditioning, acute functional decline” (p. 81, emphasis added). Hofmann and Hahn (2014) similarly describe environmental adaptations that might “reduce *risk of* injury” (p. 84, emphasis added) that are associated with routine restraint usage. The routine ways that staff use force with residents with responsive behaviors and cognitive impairments points to a critical attribute of iatrogenesis in this context: it is habituated. Environmental conditions and staff actions put residents at risk for injury not just once or rarely, but routinely. The routine nature of iatrogenesis identified in this definition does not necessarily imply that the same harms befall residents time and time again (although this is often the case), but rather that certain ways of providing care in this context have become habitual within institutional dementia care and thus disappear from staff view as exceptional acts of harm.

It is with these critical attributes in mind that we offer the following definition of iatrogenesis in residential dementia care: habituated, forceful, hands-on care provided to residents who exhibit responsive behaviors that results in emotional, physical, spiritual, social harm, and/or gradual functional decline, with the intention of supporting the resident’s safety and dignity. This definition distinguishes iatrogenesis from similar concepts, such as deliberate abuse and inadvertent harms (Table 2). It also distinguishes iatrogenesis from other common health care-related harms, such as nosocomial infections, which are not transmitted exclusively to residents with responsive behaviors and are typically the result of procedural errors rather than overt attempts to ensure safety or dignity over and above the best available evidence.

**Table 2.** Model, Contrary, and Related Cases

Case	Rationale
**Model case:** Joe is a 66-year-old resident with behavior variant frontotemporal dementia. Joe self-neglects and routinely refuses assistance with his personal care. Before the onset of dementia, he was known for his clean-cut appearance and attention to detail. Now Joe has a strong body odor and rarely consents to have his clothes changed. Joe can become very aggressive when presented with unwanted care. Staff have trialed many gentle persuasive approaches with Joe, but he finds personal care assistance intolerable despite all their efforts. Joe’s daughter is planning to visit for his birthday later this afternoon, and staff is anxious to get his care done so that his dignity is preserved. When he exits the bathroom five staff are waiting with towels and a change of clothes. Joe attempts to leave the room but is delayed by two staff who hold his hands, while the third staff member begins to undress him, and the fourth staff member begins to wash him hurriedly. The fifth staff member stands close by to intervene if necessary. Joe kicks at the staff holding him and swears repeatedly. One of the staff who is holding his hands attempts to soothe him by saying repeatedly “almost done, Joe, almost done.” Once he is washed and redressed, the staff let him leave the room. He is tearful and angry and avoids staff and his bedroom for the remainder of the shift.	In this model case, we see the critical attributes of iatrogenesis that are outlined previously. The resident presents with a responsive behavior—resistiveness to care—that staff have found has not responded to gentle persuasive approaches. Care is provided by five staff who are aware of his resistance and have provided this type of care before. They are endeavoring to preserve Joe’s dignity and have coordinated to provide care by force. This care resulted in emotional harm (anger and upset) and social harm (a loss of trust with care providers). It also puts Joe at additional risk for continued decline, insofar as he leaves the encounter angry and becomes avoidant of bathrooms entirely.
**Contrary case:** Amy is a 72-year-old resident with advanced Alzheimer’s dementia. She is entirely dependent upon the assistance of at least two unregulated care staff for her personal hygiene care and mobility, due to contractures and challenges following direction. Staff typically wash her while she is in bed. While washing her back, a staff member notes a papule on Amy’s back and begins squeezing it to excavate it. Amy thrashes in bed and screams. The accompanying staff member rushes over to hold Amy’s arms down and asks her colleague what she is doing. Her colleague replies that she cannot stand the sight of papules and that she’s getting rid of it. She asks her colleague to continue holding down Amy’s arms for just a moment longer. The staff member does so but says “I don’t know if you should be doing this. Isn’t there a policy that says this is outside of our skillset?”	This contrary case does not satisfy the four critical attributes outlined previously. The resident does not initially present with responsive behaviors, and the staff member’s intention is not to preserve dignity or safety. This form of forceful care is not habituated; the colleague questions it and draws attention to an institutional policy that is in place to prevent the evacuation of papules by unregulated care providers. While it is uncomfortable to label it as such, given how commonplace such an event might be, the emotional and potential physical harms that Amy will endure from this procedure satisfy the conditions for abuse rather than iatrogenesis.
**Related case:** Karol is a 98-year-old resident with vascular dementia who is generally quiet and easy-going. One day, while a staff member is assisting Karol with washing and dressing, a male resident inadvertently walks into her room. Karol becomes very upset and begins swearing at him. She raises her fist and attempts to lunge at him. The staff member catches her arm before she can strike the resident and she ushers him out of the room. She helps Karol resettle and process the encounter, and notices that she sustained a small skin tear on her lower arm from shear. Despite diligent wound care, this skin tear becomes infected, and Karol is prescribed an oral antibiotic. She subsequently develops a *Clostridium difficile* infection and then sepsis, and she eventually passes away with her family by her bedside.	This case is missing key elements of the definition of iatrogenesis offered above. In attempting to prevent a situation from escalating, staff intervened, and the resident was injured inadvertently. Karol does display aggression toward a coresident, but her aggression was not related to unwanted care provision. She sustained an injury that eventually, considering her comorbidities, resulted in death. This injury was sustained in service of protecting the resident, but the staff response was far from habituated. The harm that resulted from this encounter was inadvertent, rather than iatrogenic.

### Antecedents

The next step in Walker and Avant’s (2019) approach to concept analysis is to identify antecedents to the concept, which are useful for “shedding light on the social contexts in which the concept is generally used” (p. 178). The residential dementia care literature highlights systemic issues as antecedents to health care-related harms. Rather than a key attribute of the definition, the dementia care literature portrays systemic gaps and inconsistencies as the conditions that lead staff to perform inadequate assessments (Dudevich et al., 2018; Hofmann & Hahn, 2014; Travis et al., 2003), fail to recognize an “unmet need” (Cotter, 2005, p. 81), and fail to act on needs they do uncover (Chandler et al., 2018). In line with the patient safety movement, these authors argue that prevailing politico-legal systems “allow harm to occur” (WHO, 2019, para. 5) and set the stage for iatrogenesis. For example, Chandler et al. (2018) argue that people with intellectual impairments are “most vulnerable to abuses of power” (p. 466), question the actions that are legally sanctioned once a person’s capacity has been called into question. They argue that it is at a legal and policy level that people’s most basic rights are first preempted and that, while protections exist for people with cognitive impairments, work is needed to “*realise* equal rights for people with disability” (p. 466) in practice. Travis et al. (2003) note that institutional policies, at times, set the stage for health care-related harms. They argue:

If staff members and other direct care providers have never been told that causing pain, even in the course of “routine care,” is not acceptable, IDP is not likely to be monitored and treated as a legitimate pain experience. Multidisciplinary, institution-wide approaches to pain management are generally the superior way to change staff attitudes and institutional commitment toward pain management. (p. 34)

Hofmann and Hahn (2014), too, note the dearth of evidence-informed institutional policy and clinical guidelines in some institutions as the primary reason for inferior practice that results in harm. In each of these articles, the authors echo the patient safety movement by highlighting how individual practitioners would be guarded against practice deficiencies if policies and organizational cultures were different. Three specific organizational inadequacies were identified in the literature: (a) inadequate education and training, (b) misunderstandings of dementia, and (c) increasing workload and demands on staff. While they are presented separately here, it will become clear that the three antecedents are interconnected.

#### Inadequate education and training

Four of the five articles (80%) indicated that inadequate education and training contribute to the development of responsive behaviors of dementia (Cotter, 2005; Dudevich et al., 2018; Hofmann & Hahn, 2014; Travis et al., 2003). They argue that, with inadequate education and training to guide them, caregivers often fail to appreciate the meaning behind behaviors and the unmet needs of the people in their care. Cotter (2005) advocates for a stronger presence from advanced practice nurses such as Clinical Nurse Specialists in residential dementia care to teach and direct staff, as a means of reducing iatrogenic harms. Dudevich et al. (2018) argue that information-sharing across institutions may improve the care provided to older adults in general, and those with dementia. Travis et al. (2003) call for staff to be trained in “pain-free approaches to personal care” (p. 39), and Hofmann and Hahn (2014) encourage evidence-based education and training opportunities for staff to reduce restraint usage. Without adequate training in skill performance and education in evidence-informed interventions, iatrogenic damages will undoubtedly arise in the form of well-intentioned staff providing care that harms residents with unmet needs.

#### Misunderstandings of dementia

The residential dementia care literature also highlights systemic issues at the root of health care-related harms. Travis et al. (2003), for example, highlight the common misperception that people who do not express pain verbally do not feel pain. In their article on the rights of people with disabilities, Chandler et al. (2018) make frequent references to “underlying structural disadvantages” (p. 479) experienced by people with cognitive impairments. They argue that stigma and misperceptions of impairment drive the “often unregulated use of restrictive practices” (p. 466), despite care staff’s best intentions to provide dignified care. While dementia care has certainly progressed beyond the care described in early literature about residential care (e.g., Vladeck, 1980), stigma and misperceptions about dementia persist and continue to impact care (Steele et al., 2020). In some instances, even basic knowledge of dementia is lacking. In a recent study on attitudes toward dementia, Alzheimer’s Disease International found that as many as 62% of health care providers worldwide think that dementia is a normal part of aging (2019, p. 12). Dementia is still widely portrayed in the broader culture as a tragic loss of self and dignity. Metaphorically, dementia is portrayed as a “thief,” a “silent tsunami,” a “plague,” and a “time bomb” (Zeilig, 2014, p. 261). People with dementia, in turn, are described as “empty shells,” “victims,” and “burdens” (Zeilig, 2014, p. 261). Care providers are not immune to these misperceptions. Misperceptions and stigma around dementia continue to impact care, even in specialized facilities (Low & Purwaningrum, 2020). Inadequate education and training about dementia and responsive behaviors can also contribute to excessive use of force in residential care and the (albeit often unintentional) dehumanization of people with dementia (Alzheimer’s Disease International, 2019).

The person-centered care framework is widely recognized as an important guide to improving care for people with dementia (WHO, 2007). When implemented and supported by the larger institution, person-centered dementia care frameworks embolden care that affirms the continued humanity of people with dementia and combats the stigma surrounding the diagnosis (Kitwood, 1997). For example, Hofmann and Hahn (2014) note that staff in RACF “should know how to provide person-centered care to manage difficult situations” (p. 3013) without using restrictive devices like restraints or excessive force. Travis et al. (2003) encourage education to support staff in recognizing and “decoding” the causes of behavioral disturbances during care (p. 34). Cotter (2005) advocates for the development of individualized care plans, based on holistic assessments, to counter the use of restraints to manage behavioral challenges. Dudevich et al. (2018) encourage attention to “emerging evidence” (p. 14) as a means of countering stigma and iatrogenic harm.

#### Workload and institutional demands

Staffing shortages in RACFs are widespread, and staff face increasing pressures to provide high-quality care with less and less resources (Travis et al., 2003). For example, poor working conditions have been found to impact the rate of restraint usage on many units (Cotter, 2005; Hofmann & Hahn, 2014). As Travis et al. (2003) note, “there are days in clinical practice when, as the saying goes ‘you can’t see the forest for the trees’” (p. 39) and do not have an opportunity to “step back and give serious thought to what or who may be causing painful experiences for residents in long-term care settings” (p. 39). An organizational focus on task completion and routinized care places pressure upon staff to complete tasks quickly and efficiently, without deference to the individual and often complex care needs of residents with dementia (Cotter, 2005; Travis et al., 2003). Additionally, care staff may experience pressure from governing bodies, residents’ supporters, and administration to perform care to a particular standard (Chandler et al., 2018). Staff is also practicing in an increasingly litigiously inclined world, where news reports abound of elder abuse and neglect, and just under half of the general public report that they believe health care providers routinely ignore people with dementia (Alzheimer’s Disease International, 2019). The expectation that all residents’ appearances and health be maintained at a certain level, despite the distress such care may elicit, may well be a powerful antecedent for iatrogenesis in residential dementia care.

### Consequences

The next step in Walker and Avant’s (2019) approach to concept analysis is to identify the consequences of the concept. Consequences are that which arise because of the concept’s occurrence. Due to the nature of the concept under scrutiny here, the consequences of iatrogenesis are integral to its very definition. Consequences of iatrogenesis in dementia care fall into three categories: (a) physical, emotional, psychosocial, and spiritual consequences; (b) increased risk for future harm, such as functional decline; and (c) ethical consequences.

Immediate consequences of iatrogenesis in residential dementia care were highlighted by three of the five articles (Cotter, 2005; Dudevich et al., 2018; Hofmann & Hahn, 2014). They included iatrogenic outcomes such as pressure ulcers, muscle deconditioning, incontinence, increased risk of falls, functional decline, delirium, entrapment, early mortality, decreased immune response, and malnutrition. Three articles highlighted the emotional consequences of iatrogenesis in residential dementia care (Cotter, 2005; Hofmann & Hahn, 2014; Travis et al., 2003). These included emotional outcomes such as anger, fear, humiliation, frustration, loneliness, and increased responsive behaviors for the resident. Three articles also highlighted the ethical consequences of iatrogenesis (Chandler et al., 2018; Cotter, 2005; Hofmann & Hahn, 2014). All five articles highlighted that iatrogenesis places residents at significant risk for future harm (Chandler et al., 2018; Cotter, 2005; Dudevich et al., 2018; Hofmann & Hahn, 2014) by, for example, impacting their physical status and leaving them more susceptible to future disease and injury (e.g., Dudevich et al., 2018). Cotter (2005) describes the ethical consequences of restrictive practices for staff and describes the tensions that staff experience when they restrict residents’ freedoms. Hofmann and Hahn (2014) describe the seeming incompatibility of bioethical principles of beneficence and autonomy in dementia care, when staff is compelled to do “good” even when it is against the resident’s wishes. Chandler et al. (2018) are perhaps the most forceful in describing these ethical challenges and highlight ways that many dementia care practices can dehumanize residents with dementia.

### Empirical Referents

The final step in a concept analysis is to determine the empirical referents for each defining attribute of a concept. These are “classes or categories of actual phenomena that by their existence or presence demonstrate the occurrence of the concept itself” (Walker & Avant, 2019, p. 179). Defining empirical referents is a way of identifying how a person might recognize the presence of defining attributes of a concept. They are not actual tools to measure those concepts, but they can prove useful in instrument development later and can help researchers identify directions for future study.

When attributes are not overly abstract, the empirical referents and defining attributes can be identical. For example, the empirical referent for the first attribute (care of residents of RACFs with a dementia diagnosis who decline interventions) is the attribute itself. Care might include hands-on care, supervisory care, or any other therapeutic interventions indicated for the custodial, medical, or social care of a resident. Currently, the linguistic and para-linguistic characteristics of residents’ refusals of care require further study to identify the various forms they take and any linguistic patterns common across them. The empirical referent for emotional, physical, spiritual, and social harms is likely the attribute itself. The presence and extent of physical harms would be captured in incident reporting in some facilities, while the extent of more abstract harms would benefit from more precise measurement in the future (Cotter, 2005). In particular, residents with advanced disease who are unable to share the impacts of interventions directly require assistance in making these harms known (Travis et al., 2003). An empirical referent for institutional support is policies and procedures that guide practice in residential dementia care facilities, along with staff documentation about their interventions. Common practices might be gleaned from shift reports, progress notes, restraint documentation forms, and the like. Empirical referents for beneficence are more abstract, and the presence of this defining attribute might best be determined by justifications that staff offer for the care they provide, and the level of moral distress experienced by staff. As noted previously, ethical consequences of performing unwanted care abound. One way to determine beneficence in care would be to examine staff members’ subjective reports of distress after the provision of care.

## Discussion

The concept of iatrogenesis is often thought to originate in medicine. Indeed, when we first considered a concept analysis of iatrogenesis we gave some thought to whether a concept *derivation* might be more appropriate. Concept derivations seek to transpose a concept from a parent field to a related field, and then to refine the concept for use in that new field (Walker & Avant, 2019). For example, Caplan and Caplan’s (2001) work proposes to transpose the medical understanding of iatrogenesis onto social work to understand the ways that psychosocial interventions might also do significant harm. Exploring the history of the concept’s winding development and multiple meanings has, however, convinced us thoroughly that the concept has not merely been transposed from one discipline to another. There are, we believe, unique contributions to be made through analysis of this concept in the specific context of residential dementia care.

While the term “iatrogenesis” originated in psychiatric medicine, it also gained powerful traction in critical theory (Illich, 1976; Sharpe & Faden, 1998). The definition of the concept in the context of residential dementia care certainly shares similarities with medical and psychiatric definitions of the term, but its antimedical roots are just as integral to understanding its meaning in the dementia care literature. Despite its focus on adverse consequences of necessary care and its focus on client conditions, the dementia care literature has a distinctly antimedicalization flare. It focuses on the vulnerability of the resident that is imposed by the dementia diagnosis, and the harms that arise from ostensibly routine care. Iatrogenesis in residential dementia care is habituated; the staff who perform the actions are behaving habitually, in ways that do not always strike those of us on the inside of the care as a problem. The problem, of course, is that “when something becomes part of the habitual, it ceases to be an object of perception: it is simply put to work” (Ahmed, 2006, p. 131). As advocates who care deeply for and have personal connections with people living with dementia, we regret times when unwanted care was simply put to work and strive to bring iatrogenesis forward as an object of perception.

### Recommendations for Future Research

Concept analysis is a preliminary phase in theory development and as such opens many possibilities for future research. Further qualitative research is needed to explore residents’ firsthand experiences of iatrogenesis. Understanding these experiences might better guide research on the impacts of receiving nonconsensual care on the overall quality of life, therapeutic rapport, morbidity, and disease advancement. Additionally, limited research has been done about residents’ refusals of care. At this time, to the best of our knowledge of how exactly residents express refusal is primarily anecdotal (Travis et al., 2003), with limited observational data and linguistic analysis available. Additional conversation analysis, observational studies, and thematic analysis of interactions between residents and staff would be particularly useful to elucidate precisely how these refusals are negotiated in practice (O’Brien et al., 2020). Widely accepted practices in dementia care also require further study, in terms of how they become and remain habituated. There has been limited research about the texts (e.g., policies, procedures, and documentation forms) that support these practices in residential care. An institutional ethnography approach that focuses specifically on refusals of care would be beneficial for understanding how practices in dementia care continue to be legitimated.

### Recommendations for Education and Practice

A person-centered care approach that prioritizes residents’ wishes over staff routines and emphasizes the humanity and contributions of people with dementia has been shown to decrease responsive behaviors and the restrictive practices that arise as a result (e.g., Hoel et al., 2021; Janes et al., 2008; Lloyd & Stirling, 2015; Stranz & Sörensdotter, 2016; Tanner, 2017; Travers et al., 2016). This literature notes the challenges frontline nursing staff experience as they try to implement person-centered policies in practice, though. They emphasize the many ways that policies idealize everyday nursing practice and fail to consider the complexities of day-to-day care. An overarching person-centered philosophy of care is critical, but it must also be paired with educational programming that helps staff determine how to navigate complex ethical challenges. Much as staff need to learn that pain caused by personal care (iatrogenic disturbance pain) is unacceptable (Travis et al., 2003), many need to learn that restraint and an unwanted touch of people with dementia are largely unacceptable in the provision of care (Chandler et al., 2018). While there are times when restraint and unwanted touch *are* very much required to prevent injury or preserve health and dignity, those times should be carefully evaluated and planned. Staff requires support and specific processes to follow to make sound decisions about when, where, and how declined interventions ought to be offered anyway.

Institutional and environmental changes have also been identified in the literature as a primary means of reducing iatrogenesis (Cotter, 2005; Dudevich et al., 2018; Hofmann & Hahn, 2014; Travis et al., 2003). Environmental characteristics that support frontline nursing staff in individualizing and personalizing their care might include flexible care routines that allowed staff to adapt their care to each resident’s situation (Cotter, 2005; Travis et al., 2003), explicitly person-centered policies (Cotter, 2005), predictable environments with familiar sensory cues (e.g., pictures, smells, and music; Cotter, 2005), adequate and consistent pain management practices (Travis et al., 2003), and consistent staffing (Hofmann & Hahn, 2014). These interventions are also reflected in the wider literature on person-centered dementia care (e.g., Lillekroken et al., 2017; Simard, 2017; Watson, 2019).

## Conclusion

In this article, we undertook a concept analysis of iatrogenesis in residential dementia care, to explore specific attributes of health care-inflicted harm in this context. The final definition of iatrogenesis in residential dementia care proposed in this article is the causation of emotional, physical, spiritual, and/or social harm to a resident or the causation of increased risk for such harms when forceful care is provided to residents exhibiting responsive behaviors with the intention of supporting safety and dignity. Iatrogenic damages are often justified by staff but may have been avoidable if an unmet needs approach to responsive behaviors was taken. The damages often occur in the absence of person-centered care.

Our analysis shares sentiments from a critical theory that iatrogenesis is largely avoidable. Our concept analysis emphasizes our commitment to reducing unnecessary and dignity-reducing care in residential facilities for people with dementia. Despite this critical flare, though, we recognize that the actual practice of providing care to residents who exhibit responsive behaviors is complex and that there are times when the harm of standing by and doing nothing seems to far outweigh the harms of intervening with force. It is, however, critical to explore and deepen our knowledge of residents’ experiences of iatrogenic harms so that frontline care providers might make these decisions with as much evidence and gentle intention as possible.
